# Sequential antibiotic exposure restores antibiotic susceptibility

**DOI:** 10.1093/jac/dkaf350

**Published:** 2025-10-08

**Authors:** Farhan R Chowdhury, Brandon L Findlay

**Affiliations:** Department of Biology, Concordia University, Montréal, Québec, Canada H4B 1R6; Department of Biology, Concordia University, Montréal, Québec, Canada H4B 1R6; Department of Chemistry and Biochemistry, Concordia University, Montréal, Québec, Canada H4B 1R6

## Abstract

**Background:**

The prevalence of antibiotic resistance continues to rise, rendering many valuable antimicrobial drugs ineffective. Pairwise cyclic antibiotic therapy, where treatment is rapidly switched between two antibiotics, has been demonstrated *in vitro* to limit the evolution of antibiotic resistance. However, what happens when resistance inevitably evolves to one of the drugs?

**Methods:**

In this study, we perform over 450 evolution experiments to test the resilience of four proposed cyclic therapies. We use soft agar gradient evolution and ‘flat plates’ to identify resistance trade-offs that are resilient to compensatory mitigation. Resensitizations were detected by antimicrobial susceptibility assays, and their mechanistic underpinnings were elucidated via genomic and phenotypic analyses.

**Results:**

Resistance evolves readily and collateral sensitivity (CS) (where resistance to drug A leads to hypersensitivity to drug B) does not hinder the evolution of multidrug resistance and does not predict or promote resensitization. However, if resistance to drug B increases susceptibility to A, a phenomenon we term backward CS, resistance to A can be reduced or even reversed. For example, we show that *Escherichia coli* cells frequently become hypersensitive to β-lactams upon aminoglycoside resistance acquisition, due to conflicting modifications to the proton motive force and efflux pumps. We also find for the first time that polymyxin B resistance can be entirely reversed by exposure to tigecycline, through the acquisition of compensatory mutations that reduce the fitness penalty of tigecycline resistance.

**Conclusions:**

The longevity of drug cycling protocols can be significantly improved by leveraging backwards CS to resensitize cells as antibiotic resistance evolves.

## Introduction

Antibiotic resistance is associated with 4.7 million deaths every year and is projected to claim 40 million lives by the year 2050.^1,2^ As pathogens are adapting to antibiotics faster than new drugs can be developed, alternative strategies to curb resistance are necessary.^3^ One proposed strategy is to use existing drugs to design sequential or cyclic antibiotic treatment regimens with drugs applied one after the other at either defined time intervals or as resistance successively emerges.^4–6^ Rapid switching delays but does not halt the evolution of resistance, and so as resistance emerges to drug A, the cycle degrades. Proper selection of drug B is proposed to increase the time required for multidrug resistance to emerge, or even allow resensitization to A.^5,7^

**Figure 1.**
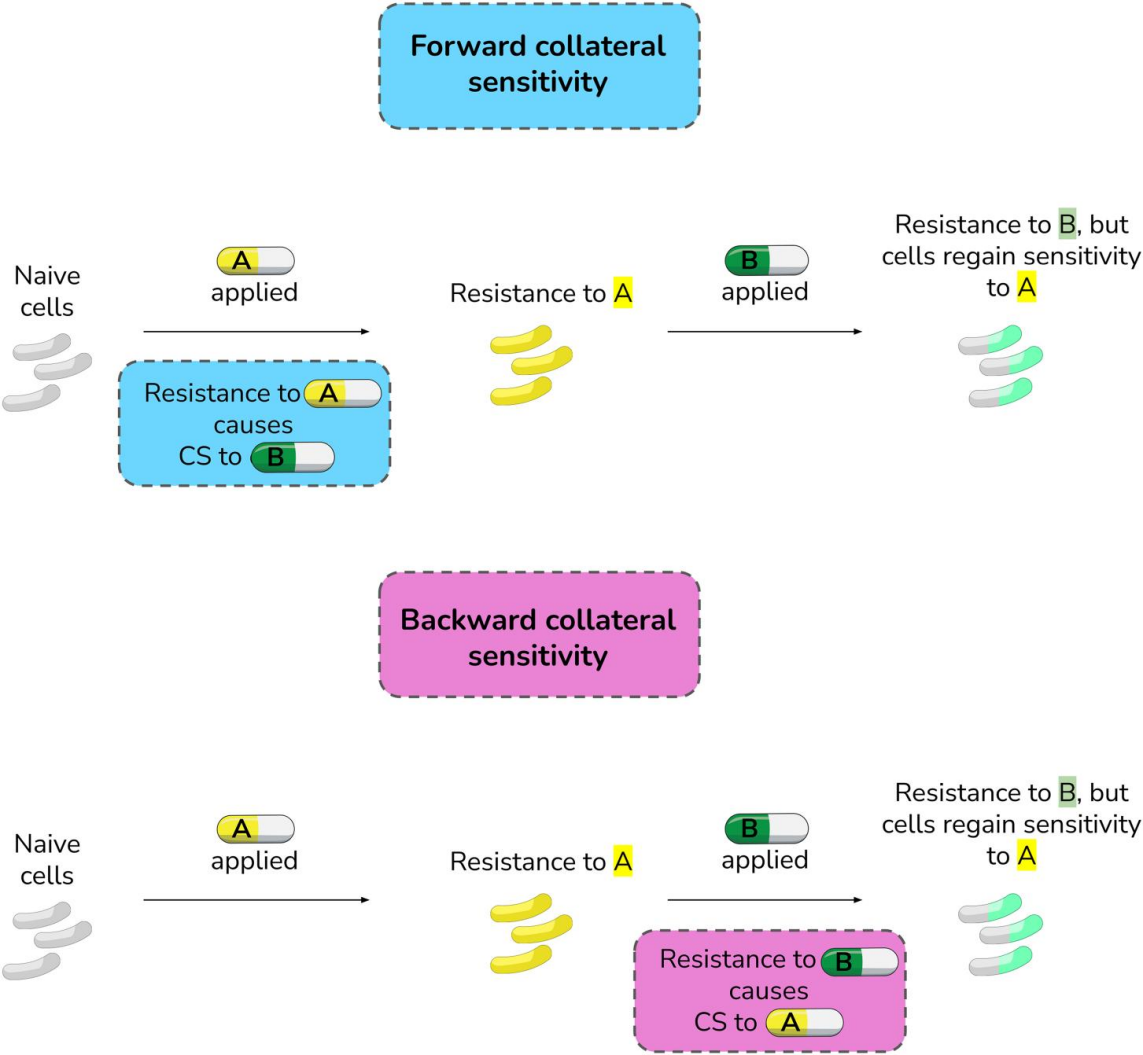
The concept of forward and backward CS.

A few studies have proposed specific drug pairs for cyclic antibiotic therapy,^8,9^ but large-scale evolutionary studies that probe the frequencies at which these pairs successfully hinder evolution or reverse resistance (resensitize) are missing. These effects must be evolutionarily repeatable to be useful in therapy.^10^ In this work, we leverage the soft agar gradient evolution (SAGE) platform^6,11^ to sequentially evolve resistance against four drug pairs proposed for cyclic therapy in 16 replicate populations of *Escherichia coli* K-12 substr. BW25113. We show that the drug pairs gentamicin–piperacillin and piperacillin–gentamicin drive 50% of the populations extinct, while the other two: ciprofloxacin–gentamicin and polymyxin B–tigecycline do not hinder resistance evolution. Upon the evolution of resistance to the second drug in the cycle the gentamicin–piperacillin pair showed no significant gentamicin resensitization, the piperacillin–gentamicin and ciprofloxacin–gentamicin pairs produced a 2-fold reduction in mean resistance to the first drug, while the polymyxin B–tigecycline pair showed a 64-fold reduction in polymyxin B resistance in every replicate population tested. To date, extinctions and resensitizations in cyclic therapy have been theoretically linked to forward collateral sensitivity (CS), where resistance to the first drug A in the cycle causes CS to the second drug B. In our data, we find no correlation between forward CS within drugs and extinctions or resensitizations. However, we find that if resistance to drug B in naive cells frequently produces CS to drug A, cells initially resistant to drug A are often rendered more susceptible to A when resistance to B emerges (Figure 1). We term this CS interaction *backward* CS (Figure 1), since CS to A evolves with resistance to B (CS direction: B to A) but antibiotics are applied in a sequence of A to B. To elaborate, consider a situation where the sequential treatment regimen is the application of antibiotic A, followed by the application of antibiotic B (treatment direction: A to B). If, via susceptibility measurements, it is determined that resistance to antibiotic A in naive bacteria induces CS to antibiotic B, we would say that this antibiotic sequence exhibits forward CS. If, instead, resistance to antibiotic B is known to induce CS to antibiotic A in naive bacteria, we would say that this antibiotic sequence exhibits backward CS. We find that backward CS helps bring down resistance levels even when bacteria sequentially acquire resistance to A and then B. We illustrate the role of forward and backward CS this by showing that in the aminoglycoside-β-lactam pair gentamicin and piperacillin, gentamicin-resistant populations exhibit widespread forward CS towards piperacillin, but subsequent exposure of cells with piperacillin CS to piperacillin does not lead to increased extinctions or significant gentamicin resistance reduction. However, when we expose piperacillin resistant bacteria to gentamicin (piperacillin–gentamicin pair; frequent CS in the opposite direction: backward CS), we see a 2-fold reduction in median piperacillin resistance. We use whole genome sequencing and efflux measurements to show that this reduction is driven by the weakening of piperacillin efflux in the gentamicin adapted strains. The effects of backward CS extend beyond the gentamicin–piperacillin pair, and we find polymyxin B–tigecycline to be a pair that highly favours resensitization to polymyxin B. Our results show the importance of considering the direction of CS and drug switching in designing effective cyclic therapies.

## Materials and methods

### Bacterial strain and growth conditions

*E. coli* K-12 substr. BW25113 and all subsequent resistant mutants were grown in Mueller–Hinton (MH) media at 37°C. Growth media were supplemented with appropriate antibiotics when growing mutants or extracting mutants from SAGE plates.

### SAGE evolutions

SAGE plates were set up to generate resistant mutants as described before.^11^ All SAGE plates were made with MH media + 0.15% agar + 0.2% xanthan gum (XAM).^12^ Antibiotic concentrations are listed in Table 1 and were determined from trial SAGE experiments to evolve strains with MICs above clinical breakpoints within 7 days. All SAGE plates were incubated for a fixed duration of 7 days. Mutants were extracted from within 1.5 cm of the end of the lanes by pipetting 20 µL of the gel into MH broth supplemented with the challenge antibiotic at a concentration = 2× the wild-type (WT) MIC (Table 2). Extracts were taken from regions with clear signs of growth. If no growth was apparent, extracts were pipetted from a random site within 1.5 cm of the end of the lane. A replicate was considered extinct if no growth was visible after overnight incubation in the antibiotic-supplemented MH broth.

**Table 1.** Antibiotic concentrations in SAGE

Antibiotic	Concentration (mg/L)
Gentamicin	5
Piperacillin	40
Ciprofloxacin	1
Polymyxin B	10
Tigecycline	5

**Table 2.** WT MICs

Antibiotic	MIC (mg/L)
Gentamicin	0.5
Piperacillin	1
Ciprofloxacin	0.0625
Polymyxin B	0.25
Tigecycline	0.25

### MIC assays

MICs were measured using the microdilution method outlined by the CLSI.^13^ Dilutions of antibiotics were prepared in MH broth and inoculated with bacteria at a final concentration = 1/200 of 0.5 McFarland standardized inoculum in non-treated 96-well plates. Plates were then incubated overnight, and the MIC was recorded as the lowest concentration of antibiotic that prevented visible bacterial growth.

### Flat plates

Flat plates were prepared as previously described.^6,14^ First, the MIC of the antibiotic that was in prior SAGE plates was determined for all strains that completed their SAGE plates. Next, we created flat lanes specific for each strain by pouring ∼12 mL of XAM supplemented with the antibiotic at a concentration = ½ the MIC of that strain in a lane of a four-well dish. This allowed maintenance of the SAGE-evolved resistance phenotype during compensatory evolution. XAM media was used for all flat plates. Plates were inoculated as described before.^11^ Each replicate passed three consecutive flat lanes. The first flat plate was incubated for 2 days and the second and the third for 1 day. The 16 gentamicin-resistant strains were used to determine the appropriate flat plate incubation times, and all three passes for these strains were incubated for 3 days instead. Extractions were carried out as described in the SAGE evolutions section, but were not limited to the 1.5 cm region of the end of the lanes. Instead, cells were extracted from where the cells had moved the farthest.

### Whole genome sequencing and analysis

Genomes were extracted from strains revived from frozen stock using the Bio Basic genomic DNA kit (cat. no.: BS624). Sequencing and variant calling was performed by SeqCenter (USA). Sequencing was performed on an Illumina NextSeq 2000, and demultiplexing, quality control and adapter trimming were performed with bcl-convert (v3.9.3). Variant calling was carried out using Breseq under default settings.^15^ NCBI reference sequence CP009273.1 for *E. coli* K-12 substr. BW25113 was used for variant calling. Figures showing common mutations in the three strains in Figure 4a and b were made using the R package *ggvenn*. For the piperacillin–gentamicin-resistant strains, mutations acquired during piperacillin adaptation were removed before analysis.

### Hexanes tolerance assay

The solvent tolerance test was performed using a protocol adapted from Ikehata *et al*.^16^ Overnight cultures for each strain were diluted 10^3^ and 10^6^ times in MH broth and 5 μL spotted on MH agar surface. Spots were allowed to air dry, and then the surface was either covered with ∼3 mm of hexanes (ACS Grade, Caledon Laboratory Chemicals, SKU: 5500-1-40) or mineral oil. Plates were sealed with parafilm and left in the fume hood to incubate for 5 days at room temperature. Plates needed refilling with hexanes every day due to evaporation.

### Antibiotic-free soft agar plates

Plates were prepared similarly to the flat plates described before but without antibiotics. Strains were inoculated on one end of these plates and were incubated for 1 day. Strains were then extracted, cultured in antibiotic-free MH broth and inoculated in a second plate. This process was repeated to achieve a total of five passes (Figure S1, available as Supplementary data at *JAC* Online). Broth cultured extracts were also streaked on antibiotic-free MH petri plates. Cells from petri plates were used to perform MIC tests.

## Results

### A SAGE-based evolution platform to test pairwise drug sequences

**Figure 2.**
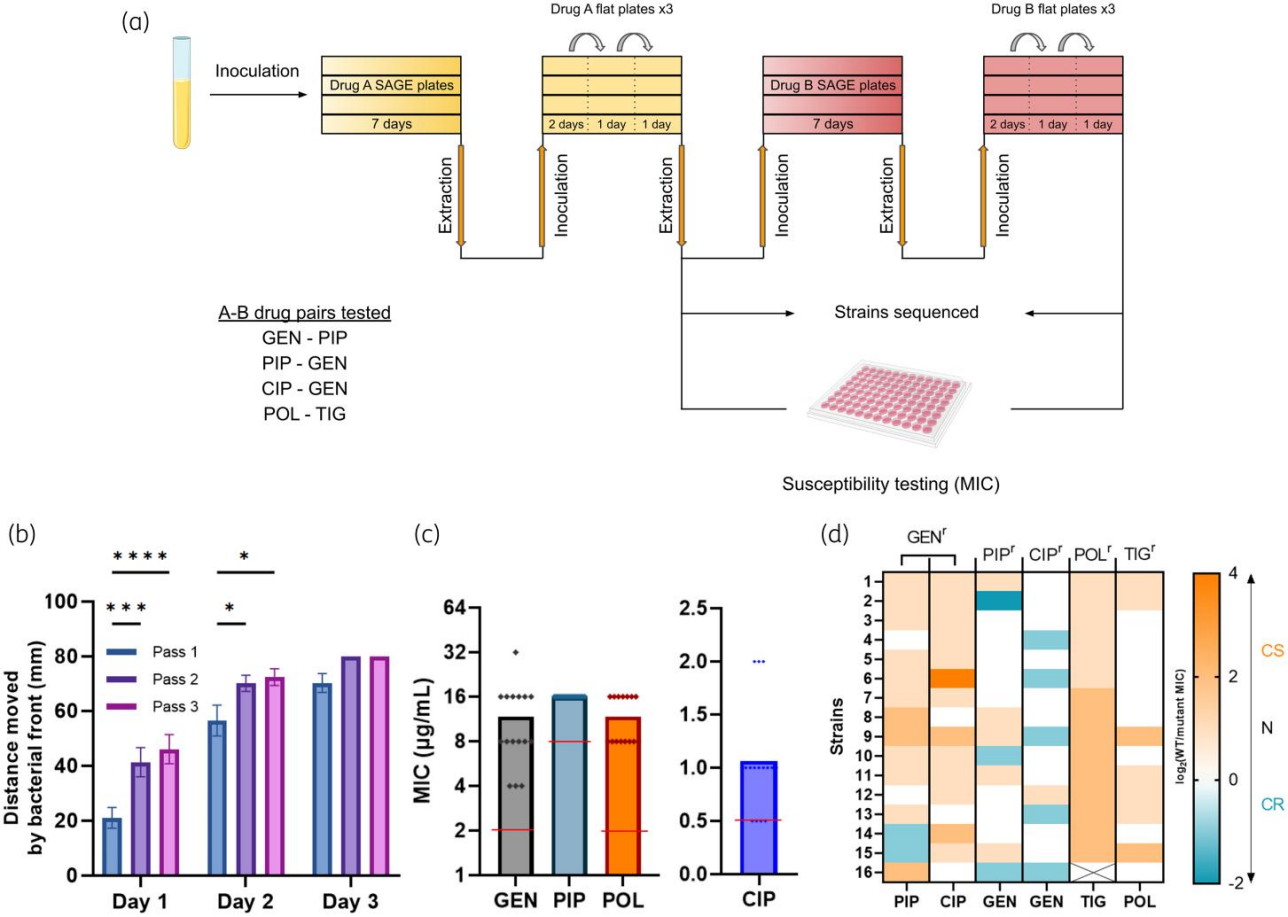
A SAGE-based evolution platform to study sequential antibiotic application. (a) Bacteria were inoculated in parallel into SAGE lanes containing antibiotic gradients and then incubated to generate resistant mutants. After 7 days, mutants were harvested and passed through three successive flat plates containing sub-inhibitory concentrations of the initial antibiotic. (b) Flat plates improve bacterial fitness in gentamicin-resistant cells, as measured by distance swam (*n* = 16). (c) Following SAGE, bacteria are resistant at or above clinical breakpoints. Horizontal lines indicate the resistance breakpoints (*n* = 15 for polymyxin B, *n* = 16 for all other antibiotics). (d) Heatmap showing CS interactions between drugs. Bacteria are resistant to the drug labelled on the top of a column, and the label on the bottom shows CS readouts towards that drug. CS and CR are shown on a log2 scale. *P < 0.05, ***P < 0.001, ***P < 0.0001, two-way ANOVA with Tukey's multiple comparisons test. GEN, gentamicin; PIP, piperacillin; CIP, ciprofloxacin; POL, polymyxin B; TIG, tigecycline.

We began with four drug pairs proposed for cyclic therapies with reported forward CS between either the drugs or the drug classes: gentamicin–piperacillin,^9^ piperacillin–gentamicin,^8,9^ ciprofloxacin–gentamicin,^17^ and polymyxin B–tigecycline.^8^ First, we used SAGE to generate 16 independent replicates of *E. coli* K-12 substr. BW25113 (WT) that were resistant to the first component of each drug pair at levels above clinical breakpoints (Figure 2a).^11^ The sole exception was polymyxin B, where we generated 15 strains. High-level polymyxin B resistance was infrequent, and to generate 15 lineages required 88 starting replicates. Next, we passed these mutants through soft agar ‘flat plates’ three times in series (Figure 2a). These plates contained the antibiotic from the prior challenge, at a concentration equal to half the MIC of the antibiotic following SAGE. We included flat plates for three reasons: (i) general growth defects like slow growth rates, common after genomic adaptation to antibiotics,^18^ can appear as false CS during MIC plate readouts,^8^ (ii) there were conflicting reports about the stability of CS,^9,19^ and (iii) we wanted to study CS interactions that are not easily reverted via compensatory mutations.^14^ We previously showed that flat plates accelerate movement of chloramphenicol-resistant strains through soft agar, significantly improving growth rates in liquid media and allowing for resistance evolution to a subsequent antibiotic at near-WT frequencies.^6^ We find here that the effect is general, with similar effects on gentamicin-resistant strains (Figure 2b). Replicates were then screened for resistance to the challenge antibiotic and for CS towards the second drug in the pair (Figure 2c and d). After SAGE evolution, the majority of the strains exhibited resistance levels above clinical breakpoints^20^ for all the antibiotics tested (Figure 2c). WT MICs are listed in Table 2.

Piperacillin CS in gentamicin-resistant replicates and tigecycline CS in polymyxin B-resistant replicates occurred frequently, with little to no cross-resistance (CR) (Figure 2d). Piperacillin resistant strains showed moderate gentamicin CS, while ciprofloxacin resistant strains showed gentamicin CS in only 1/16 replicates. Our gentamicin–piperacillin CS results reinforce previous reports that aminoglycoside–β-lactam pairs exhibit reciprocal CS,^8,9^ but some reported CS interactions, such as between ciprofloxacin–gentamicin,^17^ may either be infrequent or be mitigated via compensatory evolution.

### Forward CS does not promote extinctions, resistance drops or resensitizations in clonal populations

With a collection of strains with complete CS (polymyxin B–tigecycline), almost no CS (ciprofloxacin–gentamicin) and a mix of both CS and CR (piperacillin–gentamicin and gentamicin–piperacillin), we then evaluated whether forward CS improves extinction rates and/or promotes resistance drops and resensitizations, by subjecting each resistant replicate to the second drug in its series (Figure 2a). We considered strains as resensitized to antibiotic A when both the following conditions were met: (i) resistance drops at least 4× from prior evolved MICs and (ii) the MIC reduced to the clinical breakpoint or below. We set a strict definition for resensitization to accommodate for possible discrepancies due to random 2-fold MIC changes.^21^ Strains were considered extinct when cells could not be recovered after extraction from within 1.5 cm of the end of the SAGE plates.

**Figure 3.**
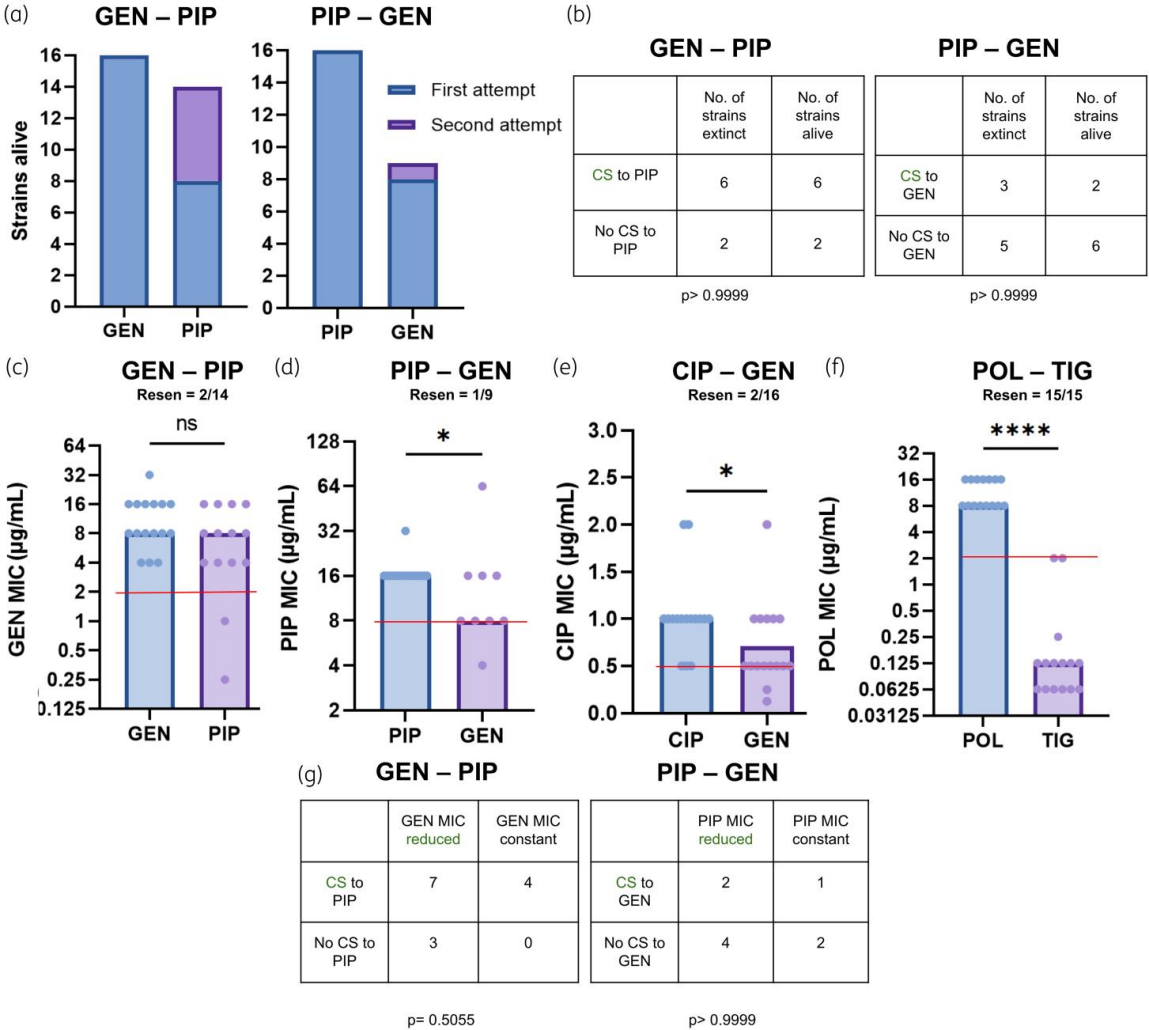
Extinctions, MIC reductions and resensitizations in drug pairs. (a) The gentamicin–piperacillin and piperacillin–gentamicin pairs cause frequent extinctions (*n* = 16 for both pairs). (b) Extinctions cannot be correlated with the incidence of CS (Fisher's exact test). (c) The gentamicin–piperacillin pair does not cause a significant reduction in gentamicin MIC (*n* = 16 after evolution to gentamicin, *n* = 14 after evolution to piperacillin). (d and e) Piperacillin–gentamicin and ciprofloxacin–gentamicin cause a 2× reduction in median piperacillin and ciprofloxacin resistance, respectively (*n* = 16 after evolution to piperacillin and ciprofloxacin, *n* = 9 after evolution to gentamicin following piperacillin and *n* = 16 after evolution to gentamicin following ciprofloxacin resistance). (f) The polymyxin B–tigecycline pair causes reliable resensitization and a large polymyxin B resistance drop (*n* = 15). Red lines indicate the resistance breakpoints. (f and g) Extinctions cannot be correlated with the incidence of CS (Fisher's exact test). Resen = resensitizations. *P < 0.05, ****P < 0.0001, Mann–Whitney test. GEN, gentamicin; PIP, piperacillin; CIP, ciprofloxacin; POL, polymyxin B; TIG, tigecycline.

We found that both the gentamicin–piperacillin and piperacillin–gentamicin pairs caused 8/16 of the replicates to go extinct (Figure 3a), even with significant differences in the prevalence of forward CS (Figure 2d). The extinctions occurred despite compensatory evolution in the flat plates, indicating a stable hurdle in adaptation to the second drug. The failures were not due to the antibiotic challenge alone, as generation of resistance to gentamicin or piperacillin in WT populations resulted in no extinctions (Figure 3a). The other two drug pairs did not show any extinctions, including the polymyxin B–tigecycline pair, which had ubiquitous forward CS (Figure 2d).

To maintain the sample size for subsequent tests with the gentamicin–piperacillin and piperacillin–gentamicin pairs, extinct replicates were re-run using the same SAGE setup. This allowed recovery of 6/8 of the extinct replicates in the gentamicin–piperacillin pair, but only 1/8 in the piperacillin–gentamicin pair, the pair with lower incidence of forward CS (Figures 2d and 3a). We found no association between the replicates that went extinct and their CS status towards drug B in the gentamicin–piperacillin and piperacillin–gentamicin pairs (Figure 3b). This suggests that factors outside CS may drive extinctions in a sequential regimen.

To test the link between CS and reduced levels of resistance we first measured drug A resistance levels after exposure to drug B in the extant replicates. The gentamicin–piperacillin pair produced no significant drop in median resistance levels following piperacillin evolution, with 2/14 replicates resensitized to gentamicin (Figure 3c). The piperacillin–gentamicin and ciprofloxacin–gentamicin pairs produced a 2-fold reduction in median drug A resistance and 1/9 and 2/16 resensitizations, respectively (Figure 3d and e). Polymyxin B–tigecycline showed a remarkable 64× reduction in median polymyxin B resistance, achieving resensitizations in all 16 replicates (Figure 3f).

Next, we looked for associations between forward CS and drug A resistance drops. However, only 1/16 ciprofloxacin resistant replicates showed gentamicin CS in the ciprofloxacin–gentamicin pair, while all polymyxin B-resistant replicates were resensitized to polymyxin B. Insufficient CS in the ciprofloxacin–gentamicin pair, and the presence of only resensitized replicates in the polymyxin B–tigecycline pair made them unsuitable for this analysis. From the gentamicin–piperacillin and piperacillin–gentamicin pairs, we found no associations between the number of strains with reduced drug A resistance and forward CS (Figure 3g). This suggests forward CS may not play a significant role in resistance mitigation in a sequential regimen when clonal populations are involved. Overall, we found that the gentamicin–piperacillin and piperacillin–gentamicin pairs can cause reliable bacterial extinctions, with 3/4 drug pairs tested producing significant drug A resistance drops. However, extinctions and resistance drops were not associated with drug B CS, and resensitizations remained low.

### Backward CS can drive resistance drops

**Figure 4.**
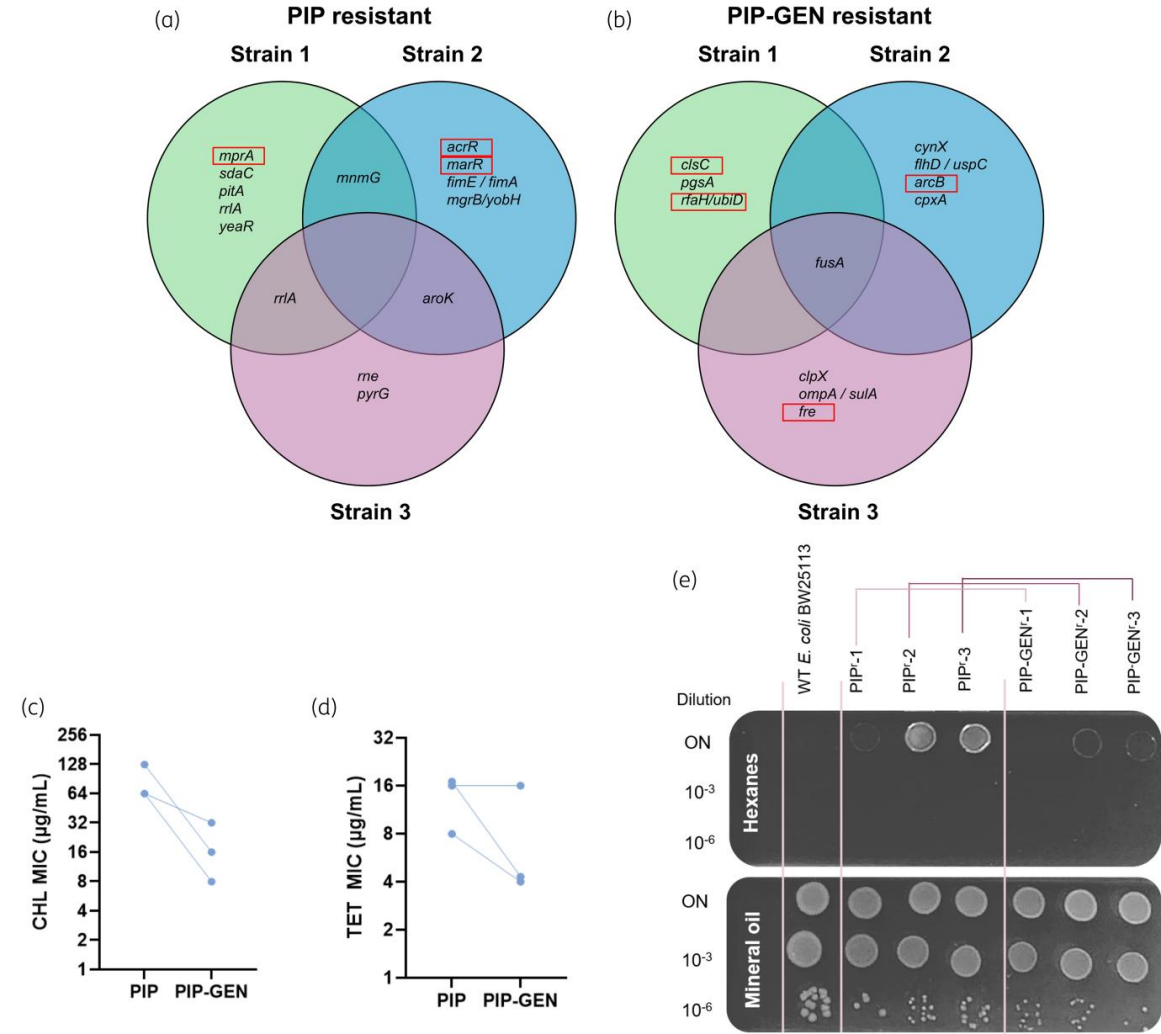
Gentamicin resistance disrupts efflux-mediated piperacillin resistance. (a and b) Mutations identified in three strains. Boxed genes are involved in efflux activity or the electron transport chain (Table 3). (c and d) Piperacillin-resistant strains are cross-resistant to chloramphenicol and tetracycline, suggesting efflux upregulation in these strains. ‘Piperacillin–gentamicin’ strains that were sequentially adapted to piperacillin and gentamicin have on average increased chloramphenicol and tetracycline susceptibility. (e) Hexanes tolerance test. All strains show good growth under mineral oil. The WT failed to grow under hexanes, while piperacillin resistant Strains 2 and 3, and to a lesser extent, strain 1, showed growth on the undiluted spots. This ability is almost entirely lost upon gentamicin adaptation, suggesting efflux disruption. Representative picture from three independent experiments. GEN, gentamicin; PIP, piperacillin; TET, tetracycline; CHL, chloramphenicol.

First, we showed that removal of antibiotic pressure does not resensitize bacteria to piperacillin or ciprofloxacin (Figures S1 and S2). To explain the mechanism that drove the resistance drops, we first looked into the piperacillin–gentamicin pair, in which 5/9 replicates showed reduced piperacillin MIC after exposure to gentamicin (Figure 3c). Gentamicin resistance is known to partially arise via mutations that weaken the proton motive force,^22^ which may disrupt the efflux-driven piperacillin resistance.^23^ To test if piperacillin resistance is driven by efflux in our strains, we first sequenced three replicates after piperacillin exposure from the piperacillin–gentamicin pair (Figure 2a). Two of the three piperacillin-adapted strains showed mutations in genes known to affect efflux: *mprA*,^24^  *marR*^25^ or *acrR*^26^ (Figure 4a and Table 3). All three strains also displayed CR to the antibiotics chloramphenicol and tetracycline (tetracycline) and the organic solvent hexanes (Figure 4c–e). Chloramphenicol, tetracycline and hexanes are all known substrates of efflux pumps in *E. coli*, indicating increased efflux capacity in the piperacillin-adapted strains.^27–29^

**Table 3.** Mutations in the piperacillin and piperacillin–gentamicin-adapted strains that affect efflux

	Piperacillin resistant	
Gene	Function	Ref.
*mprA*	Negative regulator of the multidrug transporter operon *emrAB*	^24^
*acrR*	Regulator of RND efflux pump components AcrAB	^30^
*marR*	Repressor of the multiple antibiotic resistance operon *marRAB*	^25^

To elucidate the effects of gentamicin resistance, we then sequenced three strains after gentamicin adaptation (Figure 2a). All three acquired mutations in *fusA* (Figure 4b), which codes for the elongation factor G and is known to confer gentamicin resistance.^31^ Additionally, every strain acquired mutations in genes involved in the electron transport chain: *clsC*,^32^  *ubiD*,^33^  *arcB*^34^ or *fre* (Table 4).^35^ Chloramphenicol resistance, tetracycline resistance and solvent tolerance all dropped following gentamicin adaptation (Figure 4c–e), showing that these mutations negatively affect efflux. The backward CS towards piperacillin that frequently arises with gentamicin resistance (Figure 2d, first column) may hence stem from a reduction in efflux. The median drop in piperacillin resistance levels after exposure to gentamicin also corresponds to the magnitude of piperacillin CS exhibited by WT cells resistant to gentamicin (Figures 2d, first column, and 3d).

**Table 4.** Mutations in the piperacillin and piperacillin–gentamicin-adapted strains that affect the electron transport chain

	Piperacillin–gentamicin resistant	
Gene	Function	Ref.
*clsC*	Cardiolipin synthase C supports respiratory supercomplex organization	^32^
*ubiD*	Involved in ubiquinone biosynthesis	^34^
*arcB*	Aerobic respiration control sensor protein, member of the two-component regulatory system ArcB/ArcA	^36^
*Fre*	NAD(P)H-flavin reductase, involved in transmembrane electron transfer	^37^

To test if backward CS can also explain the resistance drops in ciprofloxacin–gentamicin and polymyxin B–tigecycline pairs, we separately evolved 16 replicates against gentamicin and tigecycline to check the presence of backward CS towards ciprofloxacin and polymyxin B respectively. Gentamicin resistance imposed ciprofloxacin CS in 13/16 strains (2× CS in 10, 4× CS one and 8× in two) (Figure 2d). Efflux is important in ciprofloxacin resistance,^38^ and the efflux weakening effects of gentamicin resistance (Figure 4c–e) could also be imparting the ciprofloxacin CS. Again, the increase in ciprofloxacin sensitivity in the ciprofloxacin–gentamicin pair was equal to the magnitude of backward ciprofloxacin CS (Figures 2d and 3e, second column). Based on these results, we suggest that backward CS may disrupt resistance in A–B drug pairs, increasing drug A sensitivity. The effect of tigecycline resistance was more complex.

### Polymyxin B resensitization is multifactorial

**Figure 5.**
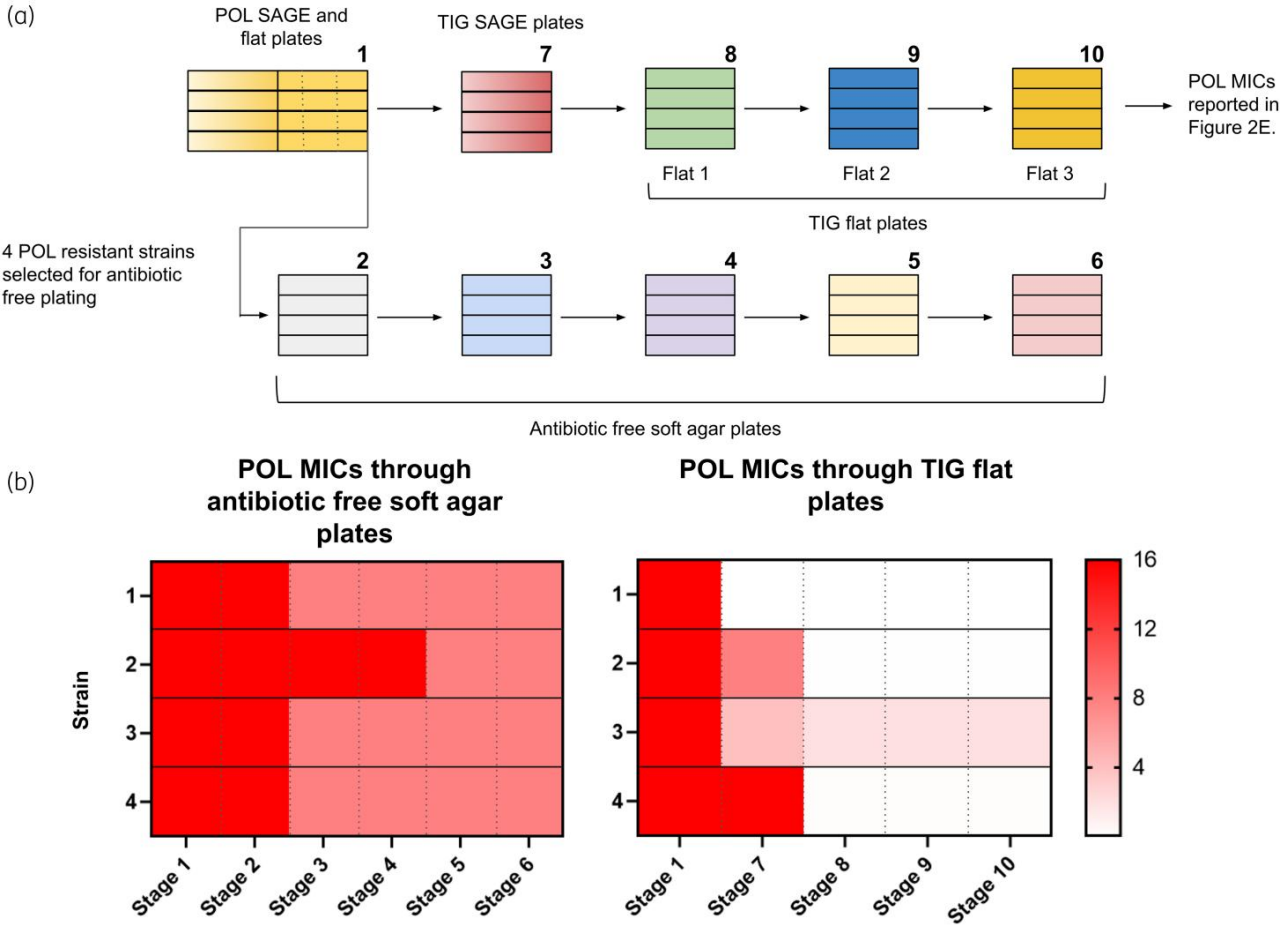
Resensitization of polymyxin B-resistant strains. (a) Scheme showing the steps of polymyxin B and tigecycline sequential resistance evolution. Numbers represent the stages at which polymyxin B MICs were performed. (b) Polymyxin B MICs at different stages of evolution. POL, polymyxin B; TIG, tigecycline.

Tigecycline resistance caused 2× polymyxin B CS in 5/16 strains and ≥4× CS in 2/16 strains (our limit of detection was 0.0625 mg/L; MIC assays with wells clear at 0.0625 mg/L were recorded as ≥4× CS) (Figure 2d, sixth column). Reciprocal CS between polymyxin B and tigecycline has been reported in *E. coli* before,^8^ but the mechanism of CS remains unknown. The resensitizations in the polymyxin B–tigecycline pair were generally stronger than the backward CS we observed, and with half of the polymyxin B–tigecycline replicates lacking CS (Figure 2d), backwards CS alone could not explain the median 64× increase in polymyxin B susceptibility. As polymyxin B MICs were measured after tigecycline SAGE evolutions and the flat plates (step 9 in Figure 5), it is possible that the change in susceptibility was due to heteroresistance or compensatory mutations.^39^ To identify the experimental stage at which the polymyxin B resensitizations occurred, we first revived four randomly selected polymyxin B-resistant strains and passaged them five times through antibiotic-free soft agar plates (Figure 5a; Figure S1). We measured polymyxin B MICs after each passage (Figure 5, 2–6) and found a maximum 2× reduction in polymyxin B MICs (Figure 5b). Next, we revived the same four lineages from frozen stocks, but this time from stages 7 to 10 (Figure 5a) and measured their polymyxin B MICs. One out of the four strains was resensitized to polymyxin B during or immediately after evolution against tigecycline, while the rest showed a 0–4× reduction at this stage (Figure 5b). Polymyxin B susceptibility in the other three strains was restored over the first tigecycline flat plate, which caused a 2–128× reduction in polymyxin B resistance. Subsequent tigecycline flat plates had no effect (Figure 5b). This suggests that cells with both polymyxin B and tigecycline resistance take evolutionary paths that promote phenotypic resistance reversion above what is achievable through the simple removal of polymyxin B selection pressure.^7,40^

## Discussion

In this study, we investigated four drug pairs proposed for cyclic therapy, generating 16 independent replicates of *E. coli* that were sequentially adapted to each drug in the pair. Owing to our large sample sizes, we were able to produce data on the reliability of these cyclic therapies, i.e. the frequencies at which they drive extinctions and resensitizations and the frequencies they fall to escape mutants that generate multidrug resistance.

We found varying degrees of extinctions in these pairs. Gentamicin–piperacillin and piperacillin–gentamicin appeared to be potent drug pairs since both the pairs drove 50% of the bacterial populations extinct (Figure 3a). However, other proposed drug pairs like ciprofloxacin–gentamicin^17^ and polymyxin B–tigecycline^8^ exhibited no extinctions, highlighting the importance of large-scale laboratory evolutions to determine drug pairs that hinder resistance.

The gentamicin–piperacillin drug pair produced no significant reduction in resistance (Figure 3c). Looking at the spread of the data, the importance of a large data set becomes clear. While a number of strains ended up losing resistance, an almost equal number of strains either retained or gained resistance, making small sample sizes vulnerable to biases.

Optimal drug pairs for cyclic therapies are thought to have been ones with forward CS.^9,17,41^ Contrary to this, we found no significant associations between forward CS and bacterial extinction or antibiotic resensitization. Evolution of resistance to gentamicin led to frequent piperacillin CS (Figure 2d), but cells with and without piperacillin CS were equally likely to have reduced gentamicin resistance following evolution to piperacillin (Figure 3g). While piperacillin exposure did render half of the lineages extinct (Figure 3a), extinction was not correlated with the presence or absence of CS (Figure 3b). In contrast, backward CS appeared to promote reduction resistance levels. Both the piperacillin–gentamicin and ciprofloxacin–gentamicin pairs had high rates of backward CS and reductions in drug A resistance following the evolution of resistance to drug B (Figures 2d and 3d and e). Our genomic analyses and efflux activity measurements showed that increased piperacillin sensitivity in the piperacillin–gentamicin pair was driven by the disruption of efflux capacity (Figure 4 and Tables 3 and 4) and may have also played a role in ciprofloxacin CS. Backward CS was present, but not identified, in a prior study of CS pairs with reciprocal CS interactions,^9^ and our work suggests that backward CS may be partly responsible for the resensitizations seen in that study.

All SAGE evolutions were conducted over a fixed period, with mutants extracted after 7 days of incubation from within 1.5 cm of the end of the plates. This design allowed us to set a fixed benchmark to report adaptation rates and extinctions, but does not allow us to comment on if the speed at which resistance evolved was affected by the presence of CS. Future studies that track movement in SAGE plates could potentially answer this question.

The polymyxin B–tigecycline pair showed a 100% rate of polymyxin B resensitization, producing an impressive 64× drop in median polymyxin B resistance levels. While backward CS was also observed in this pair (Figure 2d, first reported by Imamovic and Sommer^8^ as reciprocal CS), the magnitude of polymyxin B resistance reductions exceeded what would be expected from CS alone. Our results indicated that multiple mechanisms contributed to polymyxin B resensitization: removal of polymyxin B selection, adaptation to tigecycline and, probably most importantly, compensatory evolution in the polymyxin B and tigecycline resistant populations. Polymyxin B and colistin resistance appear frequently in the clinic via mutations in the chromosomal gene *mgrB* which negatively regulates PhoP phosphorylation,^42,43^ or via duplications of genes involved the PmrAB/PhoPQ two-component system.^44^ Gene duplications are prone to reversal which may reinstate polymyxin B sensitivity.^45^  *MgrB* mutants are known to be fitness impaired,^46^ suggesting that compensatory mutations that mitigate fitness costs may drive resensitization.^14^ These resensitizations may be especially relevant for chronic diseases in which tigecycline and either polymyxin B or the polymyxin B analogue colistin see current use,^47^ such as cystic fibrosis.^48^

Evolution of collateral phenotypes like CS is dependent on the evolution microenvironment^49^ and may therefore differ *in vivo*. Acquisition of resistance-conferring mobile genetic elements may also bypass chromosomal resistance-bound CS,^50^ while the polymicrobial nature of many clinical infections further complicates the CS landscape.^51^ Moreover, as demonstrated both in this study and by others,^52^ CS patterns may not always be repeatable. Despite these challenges, we showed that large-scale laboratory evolution can uncover consistent CS interactions. Previous work from our lab and others has shown that lab-identified, stable interactions may also appear in the clinic.^49,53^ Our findings encourage future studies to address these barriers and advance the development of reliable, CS-guided therapeutic strategies.

Taken together, we suggest that backward, but not forward, CS may play an important role in reducing resistance levels in drug pairs. The weakening of efflux mechanisms upon exposure to a second antibiotic, as seen in the piperacillin–gentamicin, and possibly the ciprofloxacin–gentamicin pair, may provide an approach for developing more effective sequential drug therapies aimed at reducing resistance. We also provide support for the idea that an aminoglycoside–β-lactam pair can frequently promote bacterial extinction. Our results highlight the importance of thorough laboratory investigation of drug pairs and of considering the directionality of CS interactions when designing sequential drug therapies to build pairs more resilient against bacterial evolution.

## Supplementary Material

dkaf350_Supplementary_Data

## Data Availability

Sequencing data associated with this work may be found under BioProject PRJNA1207050.

## Supplementary data

Figures S1 and S2 are available as Supplementary data at *JAC* Online.
