# Supplementary material for: Sequential antibiotic exposure restores antibiotic susceptibility
Source: J Antimicrob Chemother. 2025 Oct 8;80(11):3107–17. doi: 10.1093/jac/dkaf350 (PMC12630574; doi:10.1093/jac/dkaf350)
Supplement: dkaf350_Supplementary_Data [file dkaf350_supplementary_data.docx]

**Supporting Information**

**Title: Sequential antibiotic exposure restores antibiotic susceptibility**

Farhan R. CHOWDHURY^1^ and Brandon L. FINDLAY**^1,2^

^1^ Department of Biology, Concordia University, Montréal, Québec, Canada H4B 1R6

^2^ Department of Chemistry and Biochemistry, Concordia University, Montréal, Québec, Canada H4B 1R6

**Corresponding author. Email: [brandon.findlay@concordia.ca](mailto:brandon.findlay@concordia.ca)

**
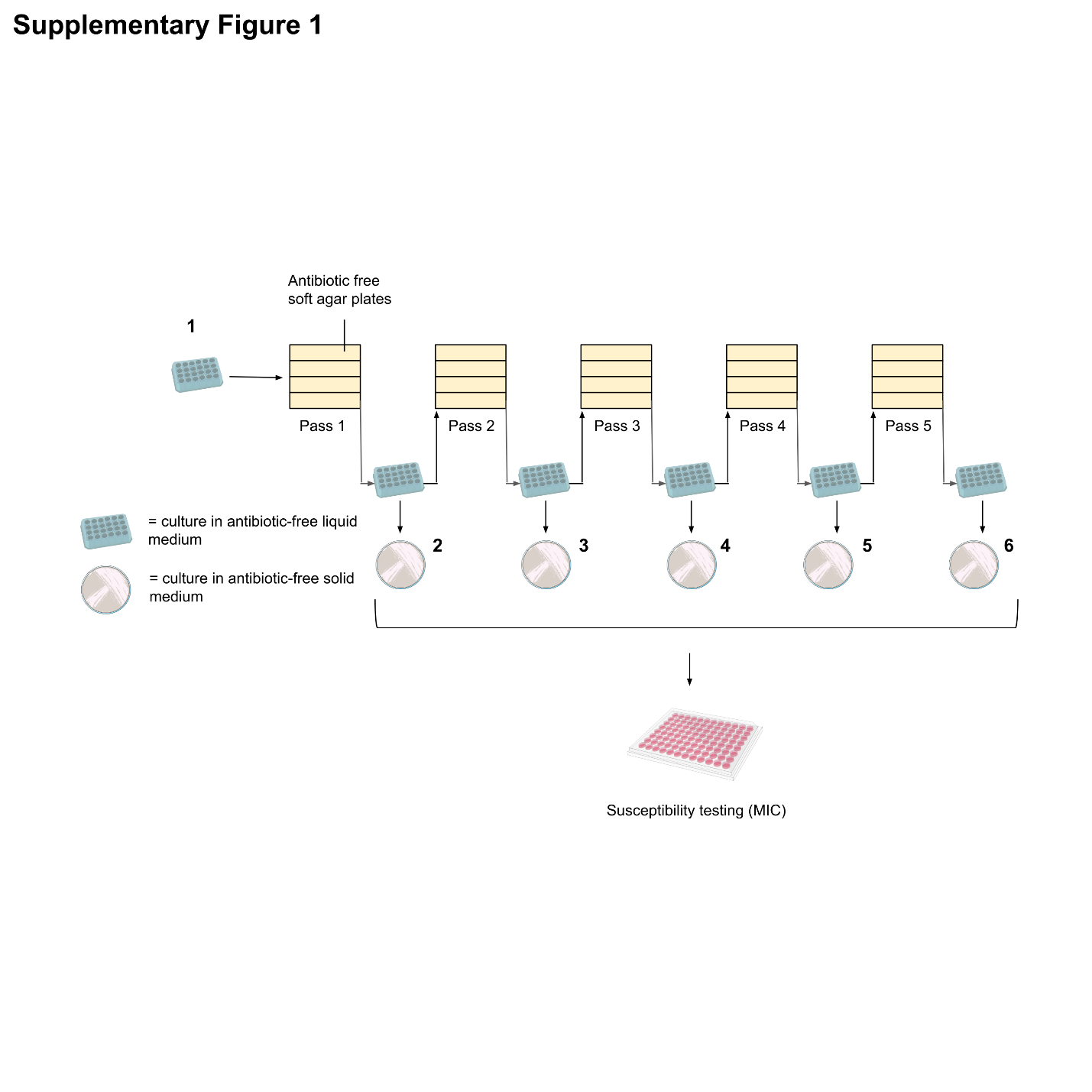
**

**Supplementary Figure 1**: Schematic for antibiotic free soft agar passages. After the 5th pass strains have undergone 5x antibiotic free soft agar passages, 5x culturing in liquid without antibiotics, and 5x cultures on antibiotic-free solid media before being MIC tested. Numbers 1-6 represent stages at which MICs were performed. Stage 1 represents MICs of strains post resistance evolution.


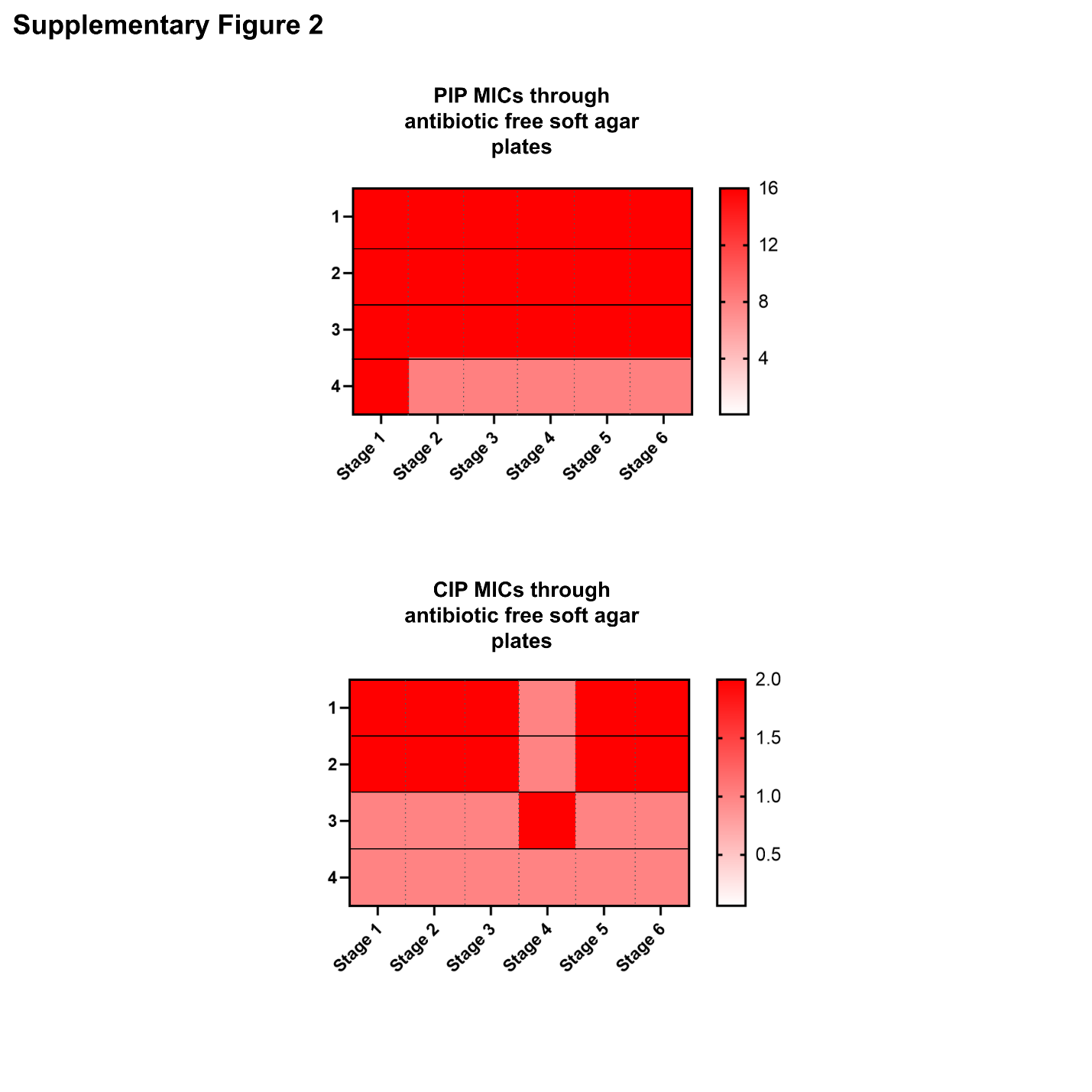


**Supplementary Figure 2**: Neither piperacillin nor ciprofloxacin MICs dropped significantly when passaged through antibiotic free medium. Passage schematic and stage numbers are on supplementary figure 1. 4 strains that showed reduced MICs from piperacillin – gentamicin and ciprofloxacin – gentamicin pairs were randomly selected for these tests. GEN = gentamicin, PIP = piperacillin, CIP = ciprofloxacin.
